# Fat Embolism Due to Avascular Necrosis Leading to Acute Chest Syndrome in Sickle Cell Disease in a Youth: A Lethal Encounter

**DOI:** 10.7759/cureus.61043

**Published:** 2024-05-25

**Authors:** Abhinav Kadam, Nikhil Pantbalekundri, Jahnabi Bhagawati, Sunil Kumar, Sourya Acharya

**Affiliations:** 1 Department of Medicine, Jawaharlal Nehru Medical College, Datta Meghe Institute of Medical Sciences (Deemed to be University), Wardha, IND

**Keywords:** hematological disorders, pulmonary complications, lethal encounter, youth, acute chest syndrome

## Abstract

Fat embolism syndrome (FES) is a rather uncommon presentation in sickle cell disease (SCD), most frequently happening in the context of long bone fractures following trauma. On the other hand, nontraumatic scenarios and nonorthopedic injuries have been documented to cause fat embolisms. This article describes the case of an 18-year-old male patient who had a known case of SCD (SS pattern). The patient complained of hip pain, and it was discovered that he had avascular necrosis of the right femoral head. The patient was started on opioid analgesics and started to respond to treatment; however, on the third day of admission, his condition deteriorated, oxygen saturation dropped, and the patient was shifted to the intensive care unit, where he was diagnosed with FES due to avascular necrosis. The patient's condition further deteriorated; he could not be saved and succumbed to death within one day. Very rarely has SCD with FES been reported in the literature.

## Introduction

Fat embolism syndrome (FES) is an uncommon event that is typically reported in conjunction with long bone fractures that are traumatic [1]. It is rarely seen in sickle cell disease (SCD) which has a significant morbidity and fatality rate [2]. Individuals with heterozygous SCD are more likely to experience this. Because FES is uncommon, it might be difficult for physicians to diagnose because it is easy to overlook. To make the diagnosis, a strong index of suspicion is necessary. Fever, fatigue, and chronic back and abdominal pain are the most typical presenting complaints of patients with imminent FES and bone marrow necrosis (BMN) [3]. These symptoms can quickly worsen and cause catastrophic multiorgan damage. The diagnosis can be made based on laboratory results such as anemia, low reticulocyte count (RC), thrombocytopenia, raised lactate dehydrogenase, high ferritin, increased immature red blood cells (RBCs), peripheral smear suggestive of leukoerythroblastosis, and magnetic resonance imaging (MRI) of the brain with diffuse microemboli in the white matter. Fever, neurological symptoms with altered mental state and petechiae, and respiratory symptoms with hypoxia and tachypnea are among the clinical findings.

Red cell exchange transfusion can potentially save lives if done as soon as FES is diagnosed [4]. Exchange transfusion enhances pulmonary vascular circulation by decreasing the viscosity and reducing inflammatory indicators and the proportion of sickle hemoglobin (HbS). Despite a rise in red cell exchange transfusion practice, overall mortality remains high. Some cases following exchange transfusion have been documented to involve therapeutic plasma exchange (TPE), which can eliminate fat globules already discharged from circulation. Chronic red cell exchange transfusion is advised for long-term care of patients with persistent symptoms.

In this case, we report a young male who was a known case of SCD and presented with avascular necrosis of the right femoral head, which was further complicated by FES during the hospital course.

## Case presentation

An 18-year-old male presented to the outpatient department of this hospital with chief complaints of back pain and hip pain for five days and fever (on and off) for two days. He had a known case of SCD “SS” pattern since birth and was taking folic acid tablet 5 mg once a day. There were no other complaints. On examination, pulse was 124/min, blood pressure was 120/70 mmHg, and blood oxygen saturation was 99% on ambient room air. Pallor and icterus were present, and there were no signs of clubbing, cyanosis, lymphadenopathy, or edema. A local examination of the right hip joint suggested pain in the joint in a full range of motion.

On systemic examination, cardiovascular and respiratory system examination was within normal limits. The abdomen examination did not reveal anything significant. The central nervous system examination was also within normal limits, except for the patient who seemed agitated due to pain. An abdomen examination did not reveal any significant findings. Laboratory investigations were sent and came back, as mentioned in Table 1.

**Table 1:** Laboratory investigations of the patient on admission g/dl: Gram per deciliter; micron: micrometer; pg: picogram; cumm: cubic millimeter; fL: femtoliter; mg/dl: milligrams/deciliter; mEq/L: milliequivalents/liter; IU/L: international units/liter; IU/ml: international units/milliliter; U/L: units/liter; mm/hr: millimeter/hour; mg/L: milligrams/liter; MCHC: mean corpuscular hemoglobin concentration; MCV:  mean corpuscular volume; MCH: mean corpuscular hemoglobin; RBC: red blood cell; WBC: white blood cell; RDW: red cell distribution width; ESR: erythrocyte sedimentation rate; CRP: C-reactive protein; APTT: activated partial thromboplastin time; INR: international normalized ratio; SGOT: serum glutamic oxaloacetic transaminase; SGPT: serum glutamic pyruvic transaminase; HIV: human immunodeficiency virus

Laboratory parameter	Results	Normal values
Hemoglobin	9.1 g/dl	11-14 g/dl
MCHC	33.2 g/dl	32-36 g/dl
MCV	88 micron	79-92 micron
MCH	29.2 pg	27-31 pg
Total RBC count	3.11 x 10^6 ^cells/cumm	2.50-5.50 x 10^6 ^cells/cumm
Total WBC count	17800 cells/cumm	4000-11000 cells/cumm
Total platelet count	2.93 x 10^6 ^cells/cumm	1.50-4.50 x 10^6 ^cells/cumm
Hematocrit	27.4%	40-54%
Monocyte	06%	2-8%
Granulocyte	63%	40-60%
RDW	18.3 fL	12.2-16.1 fL
Eosinophils	01%	1-4%
Basophil	0%	<1%
ESR	37 mm/hr	<15 mm/hr
CRP	35.683 mg/L	<8 mg/L
Urea	22 mg/dl	6.24 mg/dl
Creatinine	0.8 mg/dl	0.59-1.04 mg/dl
Sodium	140 mEq/l	135-145 mEq/l
Potassium	4.5 mEq/l	3.5-5.1 mEq/l
Alkaline phosphate	101 IU/L	75-124 IU/L
SGOT	55 IU/L	8-45 IU/L
SGPT	23 IU/L	7-56 IU/L
Total protein	9.5 g/dl	6.0-8.3 g/dl
Albumin	4.6 g/dl	3.4-5.4 g/dl
Total bilirubin	2.4 mg/dl	0.1-1.0 mg/dl
Conjugated bilirubin	0.4 mg/dl	0.1-0.4 mg/dl
unconjugated bilirubin	2.0 mg/dl	0.2-0.6 mg/dl
HIV card test	Negative	-

**Figure 1:**
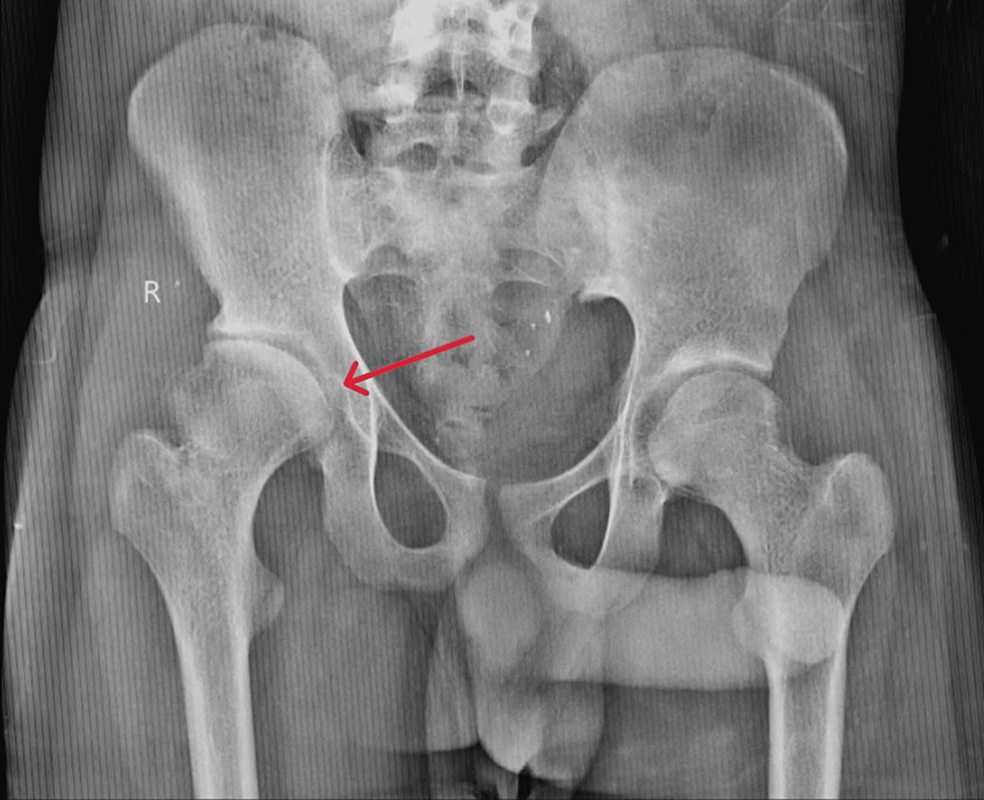
An X-ray of the pelvis and both hips showing bony irregularities in the contour of the right hip joint (red arrow)

An electrocardiograph (ECG) was done, suggesting normal sinus rhythm with no ST-T changes. An X-ray of the pelvis with both hips was done (Figure 1), suggesting bony irregularities in the contour of the right hip joint, and an MRI was advised.

**Figure 2:**
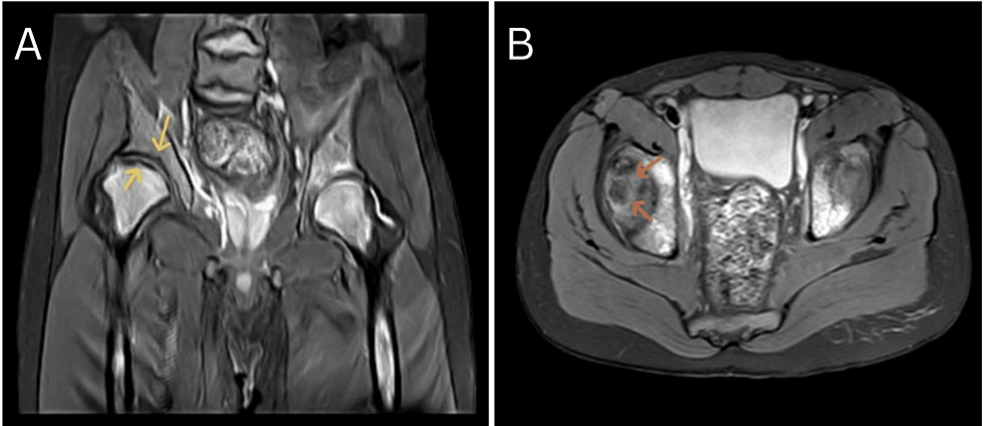
MRI of the bilateral hip joint proton density fat-suppressed image coronal section (A) and axial section (B) showing mild cortical irregularity along the superolateral part of the right femoral head and acetabulum (yellow arrows) and double line sign (orange arrows) suggesting avascular necrosis of the superolateral part of the right femoral head MRI: Magnetic resonance imaging

MRI of both hips was done, which was suggestive of avascular necrosis of the superolateral part of the right femoral head (Figure 2).

The patient was started on oral hydroxyurea 500 mg every 24 hours, a tablet of folic acid 5 mg every 24 hours, and a tablet of sodium bicarbonate 500 mg every 12 hours for the treatment of SCD. Pain management was duly taken care of with the help of nonsteroidal anti-inflammatory drugs (NSAIDs) and opioid analgesics like ibuprofen and fentanyl, respectively, as and when needed. Hydration was appropriately maintained to prevent any further sickling. The patient started responding to the treatment, the pain started to subside, and no further fever episodes were recorded.

**Figure 3:**
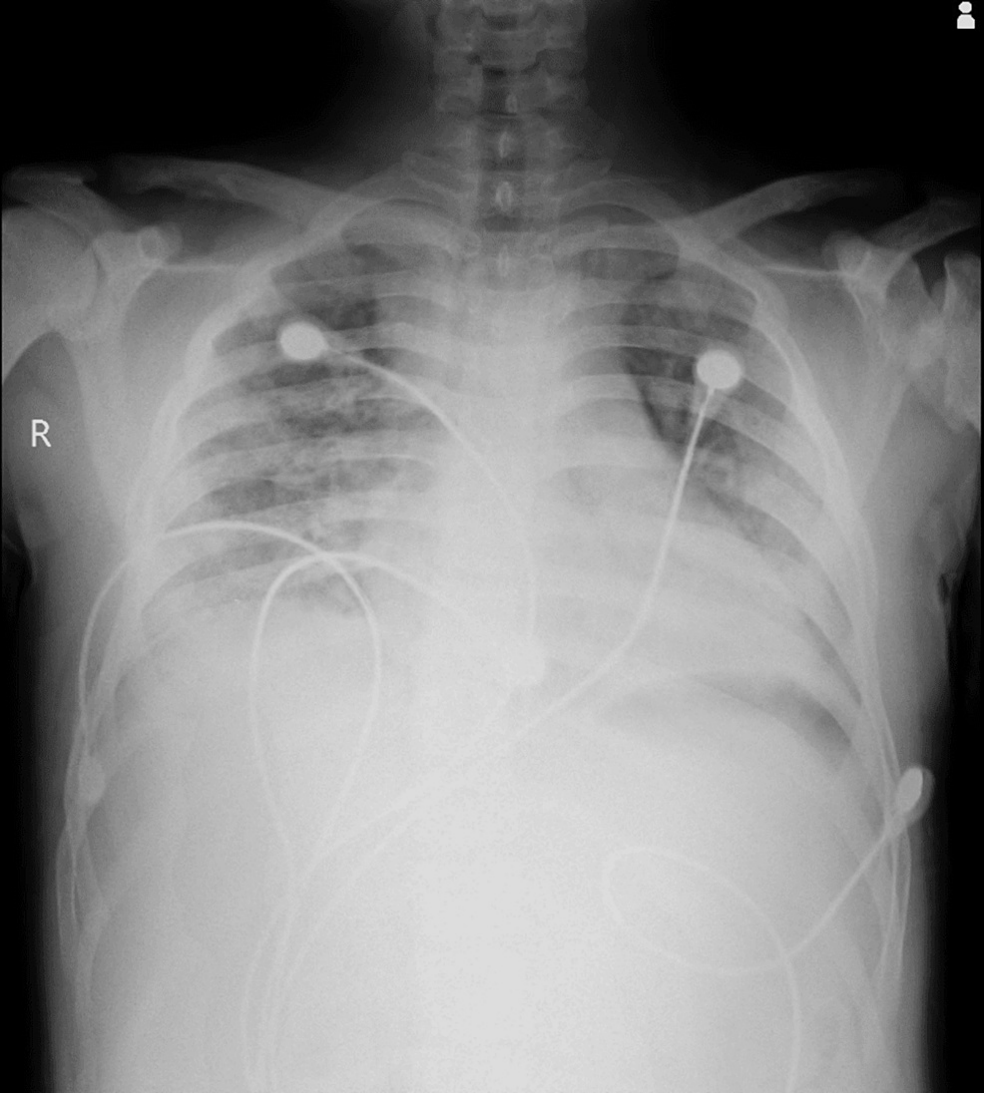
Chest X-ray of the patient on the third day since admission, suggestive of diffuse bilateral opacities throughout the lung fields

On the third day of admission, the patient developed deep icterus, and his consciousness level declined such that the Glasgow Coma Scale (GCS) went from 15/15 to 9/15. His saturation was 78% on room air, his heart rate was 144/min, and his blood pressure was 90/60 mmHg. ECG was repeated, which suggested sinus tachycardia. A chest X-ray was done, which suggested diffuse bilateral opacities throughout the lung fields (Figure 3).

The patient was immediately shifted to the medical intensive care unit. Laboratory investigations were done, which suggest increased direct and indirect bilirubin, deranged liver enzymes, and raised D-dimer levels, as shown in Table 2.

**Table 2:** Laboratory investigations of the patient on the third day since admission SGOT: serum glutamic oxaloacetic transaminase; SGPT: serum glutamic pyruvic transaminase; g/dl: gram per deciliter; IU/ml: international units/milliliter; mg/dl: milligrams/deciliter; mg/dl FEU: milligrams/deciliter fibrinogen equivalent unit

Laboratory parameter	Results	Normal values
Alkaline phosphate	238 IU/L	75-124 IU/L
SGOT	383 IU/L	8-45 IU/L
SGPT	260 IU/L	7-56 IU/L
Total protein	6.7 g/dl	6.0-8.3 g/dl
Albumin	3.0 g/dl	3.4-5.4 g/dl
Total bilirubin	8.8 mg/dl	0.1-1.0 mg/dl
Conjugated bilirubin	4.3 mg/dl	0.1-0.4 mg/dl
Unconjugated bilirubin	4.5 mg/dl	0.2-0.6 mg/dl
D-dimer	6830 mg/dl FEU	<0.50 mg/dl FEU
Calcium	8.0 mg/dl	8.4-10.2 mg/dl
Magnesium	2.1 mg/dl	1.6-2.3 mg/dl
Phosphorus	2.2 mg/dl	2.5-4.5 mg/dl

He was intubated because of low GCS and was started on inotropes; injection heparin 5000 IU every six hour and prophylactic antibiotics were started. To pinpoint the diagnosis, a computed tomography pulmonary angiography (CTPA) was planned, but the patient's condition deteriorated, and he went into cardiorespiratory arrest. He was declared dead within 12 hours of shifting into the medical intensive care unit.

## Discussion

FES is an uncommon event that is typically reported in conjunction with long bone fractures that are traumatic [1]. However, nontrauma-related causes are uncommon and include sickle cell hemoglobinopathies, osteonecrosis, liposuction, pancreatitis, cardiopulmonary bypass, osteomyelitis, and sepsis [2]. BMN is considered the etiology of FES in SCD patients. Roughly 90% of individuals with BMN have an underlying hematological malignancy as their primary etiology, with autoimmune diseases, infections, and SCD being infrequent causes [5].

FES is diagnosed based on three symptoms: axillary and subconjunctival petechiae, neurological symptoms with altered mental state, and pulmonary symptoms with hypoxemia. The symptoms of a fat embolism usually appear 24 to 72 hours after the bone injury, although they might appear as early as 12 hours after the injury. The syndrome's initial symptoms, known as pulmonary symptoms, might appear 24 hours after the triggering event and affect nearly all patients. There are several clinical criteria for diagnosing FES, but the one developed in 1974 by Gurd and Wilson is the most appropriate for our situation [6]. Our patient developed abrupt tachycardia, acute encephalopathy, fever (102°F), and acute hypoxic respiratory failure necessitating intubation.

It is still unclear how fat embolism pathophysiologically works. It is linked to two mechanisms, namely, the mechanical and biochemical hypotheses. According to the mechanical explanation, acute chest syndrome (ACES) is caused by fat globules that break free from the bone marrow, enter the pulmonary vascular bed, and create emboli in the lung during a vaso-occlusive crisis [7]. However, when the BMN is large, a significant amount of fat globules are released into the blood circulation, which causes the FES [8,9]. After that, it enters the bloodstream and forms emboli in the skin, retina, and brain. According to the biochemical explanation, phospholipase A2 breaks down embolized fat globules stuck in the lungs into harmful intermediates such as free fatty acids and inflammatory cytokines. During acute crises or trauma, high C-reactive protein induces extremely low-density lipoproteins and chylomicrons to consolidate and form fat globules. Increased catecholamines also stimulate free fatty acid synthesis, which affects pneumocytes and gas exchange right away [10,11]. It combines tissue damage from inflammatory cytokines with mechanical blockage. Human parvovirus B19 has been found to have a 24% correlation with BMN and fat embolism, which is vital for pathogenesis [12]. For unknown reasons currently, people with heterozygous, such as hemoglobin SC+ (HbSC+), rather than homozygous SCD, are more likely to experience BMN and eventual FES [12,13].

Fever, fatigue, and chronic back and abdominal pain are the most common initial complaints reported by patients with imminent BMN and FES. These symptoms, which include respiratory failure with hypoxia, tachypnea, neurological signs with confusion and diffuse infarction, and neurological symptoms with confusion, can rapidly increase and result in catastrophic multiorgan damage. Patients experiencing BMN who are in the first phases of FES are frequently mislabeled as having thrombotic thrombocytopenic purpura (TTP), as both patient subgroups have acute renal damage, encephalopathy, thrombocytopenia, fever, and anemia [14]. Peripheral smear and thrombocytopenia severity distinguish FES and TTP, while TTP has more severe thrombocytopenia. Leukoerythroblastosis is a frequently reported peripheral smear finding in FES, but schistocytes are less frequently seen than TTP [14]. However, the reticulocyte count (RC) is higher in TTP patients and lower in FES patients because of BMN [6]. Therefore, whether a patient is homozygous or heterozygous, early identification of FES can be achieved by signs or symptoms, particularly if the patient is a known case of SCD. ACES is the most prevalent cause of mortality in sickle cell patients. This is because FES is often misdiagnosed as bacterial pneumonia and treated with medicines, which are subsequently found during a postmortem analysis [15]. The majority of the FES diagnosis is made clinically. Because simple blood work can be obtained quickly, clinicians should confine their examination to blood work-ups such as arterial blood gas analysis, basic electrolytes panels, and complete blood counts with differentials to help diagnose. The most commonly reported aberrant laboratory results are a substantial rise in lactate dehydrogenase and ferritin and an acute reduction in hemoglobin and platelets. While a bone marrow biopsy can aid in identifying FES, waiting until its completion before implementing the necessary therapy might exacerbate the condition's consequences. Because of its quick recovery from acute damage, BMN cannot be ruled out by bone marrow biopsy since it depends on the timing of the test.

By reducing the amount of hemoglobin S and inflammatory indicators, red cell exchange transfusion enhances pulmonary vascular circulation by decreasing viscosity. Similar to our situation, delays in diagnosing FES are frequent and have been linked to lethal outcomes [11]. Tragically, the identification of hemoglobinopathies and other non-traumatic causes of FES is frequently overlooked in patients, particularly if the nontraumatic reasons were not previously identified in the patient's medical history. Since long bone fractures are the leading cause of FES, early orthopedic intervention is recommended in those cases. Only FES caused by hemoglobinopathies can benefit from exchange transfusions or repeated RBC transfusions; these treatments can save a patient's life and should begin as soon as possible once a patient is diagnosed with FES [11,13].

Since there have only been a few previous case reports or case series describing the appearance of extensive, bilateral fat emboli in the pulmonary vasculature in SCD following avascular necrosis of the femoral head. In our case, such extensive fat emboli involved liver and lungs, causing acute hepatitis and sudden demise of the patient. Previous reports of hepatitis in SCD due to various causes has been documented in the literature [16]; however, more published research is required before this can be adopted as a possible complication during management of patients in routine practice.

## Conclusions

In conclusion, we covered the uncommon presentation of hemoglobinopathy-related FES, which can be fatal if not identified promptly. To diagnose and take prompt action for FES, a high index of suspicion is required. For physicians, diagnosing an FES in hemoglobinopathies is difficult, particularly in cases where hemoglobinopathy has not been diagnosed. Raising awareness is necessary to reduce morbidity and death from the related multiorgan damage and to ensure an early diagnosis and start of therapy.
